# Communications

**Published:** 1902-06

**Authors:** 


### COMMUNICATIONS.

### AN INTERESTING PECULIARITY.

PITTSFIELD, MASS., June 5, 1902.

W. HORACE HOSKINS, Editor,

DEAR SIR: A little while ago I noticed for the first time a physical peculiarity which is constant in our domesticated animals of the ox tribe—a peculiarity that, so far as I know, has not hitherto been noticed or published by anyone, and which, although perhaps only a matter of curiosity, may yet be of interest to your readers. The peculiarity to which I refer is the following:

The long hairs which form the tuft at the end of the tail of the ox (generie) are invariably twisted or curled, and this twisting takes place uniformly in one direction, viz.: from right to left, and never from left to right. These hairs are sometimes twisted up into one large rope-like curl, but more frequently they arrange themselves in a mass of curls; but, whether in one large curl or in multiple curls, the spiral arrangement is invariably in the direction indicated. Observation of many hundred heads (or tails) of cattle without finding an exception seems conclusive proof that the peculiarity is constant in the domestic ox. Further, in new-born calves as soon as the hair is dry the same twisting of the hairs at the tip of the tail is to be found, and by the time the animal is two weeks old they make a dainty little whorl covering the end of the caudal appendage.

Prototypes of this peculiarity of twisting in a definite direction are frequent in the vegetable kingdom. Thus the convolvulus, Passion flower, and French bean always twine from right to left; while the hop, black begonia, and honeysuckle constantly twine from left to right, but for these phenomena botanists have not offered any reasonable explanation.

As an explanation of the peculiarity as seen in the ox I would suggest the following: In ruminant animals, which live to a large extent on bulky food, the rumen or front stomach is extremely large, comprising three-fourths of the total size of all the abdominal contents. This stomach occupies the entire left half as well as a considerable portion of the right half of the abdominal cavity, consequently we find that the left flank in the ox tribe is considerably more prominent than the right. This is more especially noticeable after the animal has partaken of a full meal, and is readily seen in any herd of cows coming home from pasture, the disparity in the contour of the two flanks then being very great. Now when a cow swishes her tail, say for the dislodgement of offending flies, she swings it up on the upper part of her back, then allows it to sweep as by gravitation down over her flanks. This sweeping friction of the tail as it descends would, in the case of the left flank, tend to make it rotate from right to left, and *vice versa* in sweeping over the right flank; but the contour of the left flank being greater than that of the right, the amount of friction and rotary force imparted to the tail as it slides over it, will also be greater, and this slight influence, operating through countless generations of ancestors, may, according to the evolutionary law of alteration by environment, account for the peculiarity as we see it to-day not only in the mature animal, but also in the animal newborn. In the instance of a few buffalos which I have been able to examine I find the same arrangement obtains. In this animal, however, the tail is quite short and incapable of reaching at all over the flanks, so that in this case the creature may have inherited the peculiarity from some race of progenitors having longer tails. G. W. KINNELL.

---

### A GREATER NEW YORK INSPECTOR'S VIEWS.

HAVING read "Columella's" article in the April number of the JOURNAL, the first thing that struck me was who is Columella? Anyhow, his new and conservative plan of controlling tuberculosis of cattle is an excellent one. This is a radical departure from any plan yet suggested. It is an improvement on the Bang system in Denmark, which does not seem practical in this country. Here everything relating to cattle and dairy interest must be conducted in most places (especially those dairies on the outskirts of cities) in a limited area, and conducted so as to give the most results for the least expense. Economy is the watchword in our dairies here, regardless of results, and any one locality that takes radical measures with those dairies under their control does so at a financial and business disadvantage to those dairies, and creating advantages for the outside competitor serving milk in the same market without improving the sanitary condition of the general milk-supply.

It makes an unfair business competition to the dairies that are continually losing cattle with tuberculosis, without improving the entire milk-supply or the improvement of herds from which these dairies are supplied.

Under "Columella's" plan most of this milk from these tuberculous cows might be used in the manufacture of cheese.

What is not used this way might then be pasteurized. Pasteurized milk is not a bad milk, nor detrimental to health. It is only a poor food-supply to very young infants.

To be practical, this method of "Columella" should be a national regulation—one which would govern interstate cattle and milk-supply, and I consider it well worth the attention of our Bureau of Animal Industry. With these safeguards—a national, State, and local inspection of dairies, which could be made jointly in many cases, and much saved—we would then get nearer a pure milk-supply, and *slowly and surely* eliminate tuberculosis, and during this elimination in this way much would be saved to the Government and State and many great losses to the dairymen prevented.

It seems to me that this is the nearest we have come to a solution of this great trouble, both from a sanitary and economic standpoint, and is well worth a trial. E. B. A.

---

Denmark in ten years has doubled the exports of its hog products in amount and value. In 1898 this small country, which is about twice the size of New Jersey, exported 200,000,000 pounds of bacon and hams, or nearly one-third the exports of this country. This marvellous increase, like that which has made Denmark the dairy farm of Europe, is due simply and solely to the popular education which was established in Denmark about half a century ago in the shape of rural high schools, and which gave technical instruction in agriculture. The result is that the farming population in Denmark is the best educated in the world, so much better educated than our own that a comparison would be ridiculous. Instead of improving the education of its rural districts, Pennsylvania continues a common-school system under which many rural districts reduce their own taxation as fast as the expenditure of the State increases, so that there are many parts of the State in which no high schools are provided for farmers' children. As a consequence, our farmers are complaining of hard times, while those of Denmark find their fields a constant profit.

Dr. James A. Waugh, of Pittsburg, Pa., reports continued favorable results of argentum colloidale crede in purpura hemorrhagica.

